# MEG-RRT*: A Hierarchical Hybrid Path Planning Framework for Warehouse AGVs Using Multi-Objective Evolutionary Guidance

**DOI:** 10.3390/s26072221

**Published:** 2026-04-03

**Authors:** Qingli Wu, Qichao Tang, Lei Ma, Duo Zhao, Jieyu Lei

**Affiliations:** 1 School of Electrical Engineering, Southwest Jiaotong University, Chengdu 611756, China; wuql@csrzic.com (Q.W.); malei@swjtu.edu.com (L.M.); leijieyu_swjtu@126.com (J.L.); 2 Baoji CRRC Times Engineering Machinery Co., Ltd., Baoji 721300, China; 3 School of Electrical Engineering and Information, Southwest Petroleum University, Chengdu 610500, China; qichao_tang@163.com

**Keywords:** autonomous guided vehicle (AGV), path planning, narrow passage, multi-objective evolutionary algorithm

## Abstract

Autonomous guided vehicle (AGV) navigation in high-density warehouses faces significant challenges due to narrow aisles and complex U-shaped traps. In such environments, traditional sampling-based path planning algorithms often converge slowly and produce suboptimal paths. To solve these issues, a novel hierarchical hybrid planning framework named MEG-RRT* (Multi-objective Evolutionary Guided RRT*) is proposed in this study. The proposed MEG-RRT* integrates an optimization engine based on NSGA-II into the sampling process. It guides exploration direction away from local minima by jointly optimizing convergence efficiency and safety-related objectives. Furthermore, a geometry-aware execution layer is introduced to improve motion through narrow passages and to refine the path structure. This layer includes radar-guided steering, adaptive step-size control, and ancestor shortcut operations. Comparative experiments were conducted in simulated scenarios of complex narrow passages and high-density warehouses to verify the superiority of the proposed MEG-RRT*. In complex narrow passages, the proposed algorithm achieves a 100% success rate; it also reduces convergence time by 43.5% compared to standard RRT* and by 44.9% compared to Informed-RRT*. In warehouse environments, it generates smooth, kinematically favorable paths that are 39% shorter than those produced by RRT-Connect. These results demonstrate that MEG-RRT* balances exploration efficiency and solution optimality, making it well suited for automated logistics applications.

## 1. Introduction

The Robotic Mobile Fulfillment System (RMFS) now orchestrates the core logistics of Industry 4.0, transforming warehouses into high-speed automated environments [1,2]. Within these dense operational hubs, Automated Guided Vehicles (AGVs) must transcend simple line-following to execute robust, autonomous motion planning [3,4]. Modern storage presents a uniquely hostile geometric challenge: the workspace is a labyrinth of high-density storage racks, narrow corridors, and dynamic obstacles that create unpredictable U-shaped dead-ends [5]. Success in this domain requires not only finding a path, but identifying a trajectory that navigates constrained bottlenecks with minimal computational latency and high reliability [6,7].

Sampling-based algorithms, particularly the Rapidly exploring Random Tree (RRT) [8] and its optimal variant RRT* [9], dominate the literature for high-dimensional planning, yet falter in these restricted settings [10]. The failure comes from the probabilistic mechanics of uniform sampling. Finding a route through a narrow aisle requires placing a sample exactly within the bounds of the corridor, an event with a probability close to zero when the volume of the corridor is negligible compared to the total free space [11,12]. This geometric imbalance forces the planner into “sluggish convergence”, generating thousands of redundant nodes before stumbling upon a viable passage [13]. Although heuristics like Informed-RRT* [14] attempt to mitigate this by restricting the search to a hyper-ellipsoidal subset, they introduce a circular dependency. The algorithm requires an initial feasible solution to define the informed set; in narrow-passage environments, discovering that the first solution is often the primary computational bottleneck, rendering the informed strategy impotent during the critical exploration phase [15].

The topological complexity further exacerbates these sampling inefficiencies. U-shaped or concave obstacle configurations notoriously trap standard greedy heuristics, causing local planners to stagnate in local minima [16]. Bidirectional strategies, such as RRT-Connect [17], attempt to bypass this by growing trees from both endpoints, but often produce jagged suboptimal paths that defy the kinematic constraints of AGVs. Hybrid methodologies that integrate evolutionary logic, such as Genetic Algorithms (GA) [18] coupled with RRT, offer a mechanism to improve population initialization [19]. However, existing implementations predominantly collapse optimization into a single weighted cost function [3]. For example, Zhao et al. [20] proposed a hierarchical framework for robot path planning in dynamic environments. They defined a weighted cost function that combines key goals, including obstacle avoidance and path length, into a single objective. Then, they applied an improved Gray Wolf Optimizer (IGWO) to minimize this objective, resulting in efficient global path planning. This reductionist approach obscures the nuanced trade-off between path efficiency and navigational risk, failing to distinguish between a route that is simply short and one that is geometrically robust enough to traverse a bottleneck without collision.

Furthermore, path planning for industrial AGVs is closely associated with broader operational challenges, including multi-objective decision-making and dynamic uncertainties. Recent advances in related autonomous domains provide valuable insights and strong motivation for this study. For example, the trade-off between conflicting objectives, such as navigational safety and energy efficiency, has been systematically investigated using Economic Model Predictive Control (EMPC) in marine vehicles [21]. This line of research highlights the importance of developing planning frameworks that can explicitly balance safety margins and path optimality, which directly motivates the dual-objective formulation adopted in this work. In addition, in complex logistics scenarios, advanced evolutionary approaches, such as hybrid multi-population genetic algorithms, have demonstrated strong effectiveness in multi-UAV task assignment problems [22], underscoring the capability of genetic algorithms to escape local optima in highly constrained search spaces. For multi-robot coordination, efficient performance evaluation and scheduling algorithms are essential for ensuring timely task execution under strict deadlines [23], which in turn places high demands on the convergence speed of the underlying path planners. Moreover, as automated environments become increasingly complex, addressing observation uncertainties and moving obstacles has become a critical issue, and advanced initialization strategies such as warm-start cross-entropy have shown notable computational advantages [24].

Motivated by these state-of-the-art developments, this paper presents a hierarchical hybrid path planning approach for AGVs. It combines multi-objective evolutionary logic with a geometrically aware sampling mechanism to achieve robust navigation in constrained environments. The main contribution of the work is summarized as follows:

(1) Aiming at the conflict between path efficiency and navigational risk in unstructured environments, a hierarchical planning framework is established. The Non-dominated Sorting Genetic Algorithm II (NSGA-II) [25] is embedded directly into the global guidance layer to solve the multi-objective optimization problem. By formulating “Distance to Goal” and “Failure Count” as distinct Pareto objectives, the model empowers the planner to make intelligent trade-offs, favoring longer but safer routes in open spaces while accepting higher-risk trajectories only when essential to penetrate narrow aisles.
(2) In order to resolve the geometric deficiencies of standard sampling-based methods, three novel mechanisms are introduced at the local execution level. Firstly, a “radar steering” strategy is proposed to actively scan the local topology and align expansion steps with corridor axes, effectively guiding the tree into narrow gaps. Secondly, an “ancestor shortcut” mechanism is implemented to execute dynamic topological rewiring during tree growth, thereby eliminating oscillating paths. Thirdly, “dynamic objective saturation” is developed to prevent stagnation in concave traps by adaptively flattening the potential field heuristic upon detection of repeated failures.
(3) Extensive simulation experiments are performed to evaluate the performance of the proposed hierarchical architecture in a complex narrow passage and high-density warehouse environments. The proposed approach is benchmarked against state-of-the-art algorithms, including standard RRT*, Informed-RRT*, and RRT-Connect. The results suggest that the proposed method significantly outperforms peer competitors in terms of convergence speed and success rate, particularly in scenarios characterized by complex narrow bottlenecks, verifying the effectiveness of the proposed strategies.

The article consists of five sections including introduction (Section 1). Section 2 provides the background, defines the optimal path planning problem and reviews RRT* and NSGA-II. Section 3 details the proposed MEG-RRT* algorithm, including its hierarchical framework, evolutionary guidance, and geometry-aware execution mechanisms. Section 4 presents the experimental results and analysis, comparing the performance of MEG-RRT* against benchmark algorithms in a complex narrow passage and warehouse logistics scenarios. Section 5 summarizes the conclusions and provides directions for future research.

## 2. Background

### 2.1. Problem Formulation

In this study, we address the optimal path planning problem for an AGV operating in a constrained warehouse environment. The AGV is modeled as a rigid body that moves in a two-dimensional workspace, $W\subset R^{2}$. The configuration space (C-space) denoted as C, represents the set of all possible transformations of the AGV.

**Definition 1** **(Obstacles and Safety).** *The workspace is populated with a set of static obstacles $O_{\mathrm{obs}}\subset W$, representing warehouse structures such as shelves and pillars. The obstacle region in the configuration space is defined as $C_{\mathrm{obs}}$, and the collision-free space is denoted as $C_{\mathrm{free}}=C\setminus C_{\mathrm{obs}}$. To ensure operational safety, a safety margin δ>0 is imposed around obstacles. A configuration q∈C is considered valid if and only if the minimum distance between the AGV and any obstacle is strictly greater than δ:*

(1) $$C_{\mathrm{safe}}\;=\;\left\{q\;\in \;C_{\mathrm{free}}|\mathrm{dist}\left(q,\;C_{\mathrm{obs}}\right)\;>\;\delta \right\}.$$

**Definition 2** **(Narrow Passage).** *The warehouse environment is characterized by the presence of narrow passages. A narrow passage is formally defined as a subset of $C_{safe}$ where the local clearance is small relative to the dimensions of the robot, significantly reducing the probability of sampling a valid configuration using uniform distribution.*

**Definition 3** **(Feasible Path).** *Let $q_{\mathrm{start}}\;\in \;C_{\mathrm{safe}}$ be the initial configuration and $q_{\mathrm{goal}}\;\in \;C_{\mathrm{safe}}$ be the target configuration. A path is defined as a continuous function σ:[0, 1]→C, such that $\sigma \left(0\right)\;=\;q_{\mathrm{start}}$ and $\sigma \left(1\right)\;=\;q_{\mathrm{goal}}$. A path is feasible if $\sigma \left(\tau \right)\;\in \;C_{\mathrm{safe}}$ for all τ∈[0, 1].*

The objective is to find an optimal route $\sigma^{*}$ that minimizes a cost function J(σ). In the context of warehouse logistics, the primary metric is Euclidean path length, defined as follows:

(2) $$J\left(\sigma \right)=\int_{0}^{1}{∥\sigma^{\prime }\left(\tau \right)∥}_{2}\;d\tau .$$

here ${∥\cdot ∥}_{2}$ denotes the Euclidean norm.

Thus, the optimal path planning problem is formulated as follows:

(3) $$\sigma^{*}={\mathrm{argmin}}_{\sigma \in \Sigma_{\mathrm{feasible}}}J\left(\sigma \right),$$

where $\Sigma_{\mathrm{feasible}}$ represents the set of all collision-free paths connecting $q_{\mathrm{start}}$ to $q_{\mathrm{goal}}$ subject to the safety margin δ. Finding $\sigma^{*}$ in spaces containing narrow passages and non-convex obstacles (traps) is widely recognized as computationally challenging, necessitating more robust sampling strategies.

### 2.2. Overview of RRT*

The RRT* is a sampling-based motion planning algorithm that extends the classical RRT framework by providing asymptotic optimality. The original RRT algorithm prioritizes rapid exploration and probabilistic feasibility. However, the RRT* ensures that the cost of the best path found converges almost surely to the global optimum as the number of samples tends to infinity by introducing an iterative refinement mechanism.

The standard RRT* incrementally constructs a tree T=(V, E) rooted at the initial configuration $q_{\mathrm{start}}$. At each iteration, a configuration $q_{\mathrm{rand}}$ is randomly sampled from the free configuration space $C_{\mathrm{free}}$. The algorithm identifies the nearest vertex $q_{\mathrm{nearest}}\in V$ according to a chosen distance metric. The steering function then extends from $q_{\mathrm{nearest}}$ to $q_{\mathrm{rand}}$ with a fixed step size η, resulting in a collision-free candidate configuration $q_{\mathrm{new}}$. The distinguishing feature of RRT* lies in its cost-aware tree expansion and topological update strategy, which is realized through two tightly coupled operations:

- ChooseParent: In contrast to the nearest-neighbor connection strategy, the RRT* considers a set of neighboring vertices within a radius *r* that scales with the number of samples. Among these candidates, the algorithm selects the vertex that minimizes the cumulative cost-to-come to $q_{\mathrm{new}}$ as the parent, thus allowing optimal local connections during tree growth.
- Rewire: Following the insertion of $q_{\mathrm{new}}$, the algorithm examines whether routing existing neighboring vertices through $q_{\mathrm{new}}$ can reduce their respective path costs. If a lower-cost connection is identified and is collision-free, the tree topology is updated accordingly. This rewiring process enables the continuous improvement of previously discovered paths and forms the core mechanism that enables asymptotic optimality in RRT*.

The RRT* provides strong theoretical guarantees in terms of probabilistic completeness and asymptotic optimality. However, its performance is often constrained by the use of uniform random sampling in practice. In environments characterized by narrow passages or densely cluttered obstacles, the probability of generating valid samples that successfully traverse critical bottlenecks is low. This leads to inefficient exploration and slow convergence toward high-quality solutions. In addition, the standard steering function in RRT* is inherently local and geometry-agnostic. It does not explicitly account for obstacle boundaries or corridor structures, which often results in invalid extensions that terminate prematurely due to collision constraints. This lack of geometric awareness further degrades planning efficiency in constrained regions, where successful expansion depends on coordinated sampling and informed directional guidance.

These limitations indicate that RRT* provides a strong foundation for optimal motion planning, yet its baseline formulation is inadequate for complex, structured environments. This motivates the incorporation of additional mechanisms to guide sampling and extension more effectively.

### 2.3. Overview of NSGA-II

The NSGA-II is a foundational framework for multi-objective optimization that simultaneously addresses multiple conflicting objectives without reducing them to a single scalar objective. It maintains a population of candidate solutions that is iteratively evolved toward the Pareto-optimal front. This front is composed of non-dominated solutions, where improvement in one objective necessarily leads to degradation in at least one other.

The algorithm is built upon two tightly coupled mechanisms: fast non-dominated sorting and crowding distance assignment. In fast non-dominated sorting, the population is partitioned into a sequence of Pareto fronts according to dominance relations. The individuals in the first front are non-dominated within the population and represent the best trade-offs achieved in the current generation, while subsequent fronts contain progressively inferior solutions in terms of dominance rank. This hierarchical ranking explicitly biases the evolutionary search toward convergence to the Pareto-optimal set.

The crowding distance metric is employed in NSGA-II to balance the convergence pressure by promoting solution diversity. It estimates the local density of solutions in the objective space, thereby preventing the loss of population diversity. During environmental selection, the solutions are compared based on a hierarchical criterion: a lower dominance rank is preferred; and among solutions with identical rank, those with larger crowding distances are favored. This mechanism promotes a well-distributed approximation of the Pareto front and reduces premature convergence caused by genetic drift.

In the context of robot path planning, these two mechanisms naturally correspond to the fundamental tension between exploitation and exploration. The Non-dominated sorting induces a preference for candidate paths exhibiting superior cost trade-offs, such as shorter length, lower energy consumption, or reduced clearance violation. This preference promotes greedy exploitation toward the goal. In contrast, the crowding distance criterion preserves structurally diverse path hypotheses. This property is critical for robust exploration in narrow passages, cluttered environments, or regions prone to dead-ends. The NSGA-II balances goal-directed optimization with exploratory robustness by explicitly maintaining multiple high-quality yet diverse parent nodes. This mechanism helps alleviate the premature convergence behavior commonly observed in conventional sampling-based planners.

## 3. The Proposed Algorithm: MEG-RRT*

A hierarchical hybrid planning framework, termed Multi-objective Evolutionary Guided RRT (MEG-RRT*), is proposed to address the challenges of narrow passage navigation and local minima entrapment in warehouse environments. The proposed architecture establishes a tight integration between global search guidance and local geometric exploitation. In contrast to conventional approaches that treat evolutionary algorithms and sampling-based planners as sequential or loosely coupled modules, MEG-RRT* embeds a multi-objective evolutionary engine directly into the iterative sampling loop of RRT*. The algorithm is structured into two coupled levels:

- An NSGA-II-based sampling module maintains a dynamic archive of elite nodes, jointly optimizing convergence toward the goal and exploratory viability as quantified by extension failure statistics. This layer biases the sampling distribution away from dead-end regions and toward topologically promising frontiers.
- A geometry-aware execution layer designed to resolve geometric deficiencies through four synergistic mechanisms: Radar Steering, Adaptive Step-size, Ancestor Shortcut, and Dynamic Objective Saturation.

From an algorithmic perspective, the proposed MEG-RRT* differs fundamentally from existing sampling-based methods because of its closed-loop architecture. Standard RRT* relies on uniform random sampling, which lacks geometric awareness and often leads to slow convergence in narrow passages. Although Informed-RRT* improves search efficiency by constraining the sampling region to a hyper-ellipsoidal subset, this strategy can only be activated after an initial feasible path has been found. In complex bottleneck environments, however, obtaining such an initial path remains a significant computational challenge.

To address these limitations, MEG-RRT* introduces a tightly integrated framework that connects global guidance with local execution through a dynamic failure-feedback mechanism. Specifically, the global NSGA-II engine not only generates candidate samples, but also adaptively updates the viability objective of elite parent nodes. This objective is defined as the cumulative failure count and is adjusted using real-time collision feedback from the local geometry-aware execution layer. This bidirectional information flow enables the global search to avoid topologically stagnant regions, such as U-shaped traps, while allowing the local layer to exploit the kinematic inertia inherited from the global elite archive to traverse narrow corridors more effectively.

### 3.1. Main Procedure of the MEG-RRT*

The core execution flow of MEG-RRT* is designed to balance probabilistic completeness with heuristic efficiency. Three sampling strategies are incorporated into the algorithm, including goal-biased sampling, evolutionary-guided sampling, and uniform random sampling, and are selected according to a probabilistic switching scheme.

At each iteration, a sampling source is selected through a hierarchical probabilistic mechanism. With probability $P_{\mathrm{goal}}$, a goal-biased sampling strategy is applied to promote directed exploration towards the target configuration. Otherwise, the evolutionary branch is activated according to the rate γ (detailed in Section 3.2). When the evolutionary branch is selected, a candidate state $q_{\mathrm{rand}}$ is generated by the evolutionary sampling routine. The generation process is biased by the local inertia of high-quality elite nodes, defined as the predominant growth direction inferred from the parent’s lineage. In all remaining cases, uniform random sampling is used to maintain global exploration coverage.

In addition, the extension phase employs a radar-guided steering mechanism coupled with adaptive step-size control (detailed in Section 3.3). In contrast to standard steering strategies based on a fixed step size, the proposed mechanism performs a systematic scan of the local angular space around $q_{\mathrm{nearest}}$. For each candidate direction, the maximum collision-free step length is computed using a binary search procedure. This design promotes tree expansion aligned with the corridor axis while maximizing the achievable extension length. After a successful extension, a deep topological optimization is performed using an ancestor shortcutting operation to eliminate redundant path segments.

Finally, the feedback loop is closed by updating the elite archive. This step incorporates the dynamic objective saturation mechanism. The algorithm monitors the global state variable PathFound to assess whether a feasible solution has been obtained. When no valid path exists, the distance-based objective is adaptively flattened. As a result, the optimization process places an increased emphasis on the failure count objective, facilitating escape from local minima. The detailed procedure is described in Algorithm 1.
**Algorithm 1:** Main procedure of MEG-RRT*
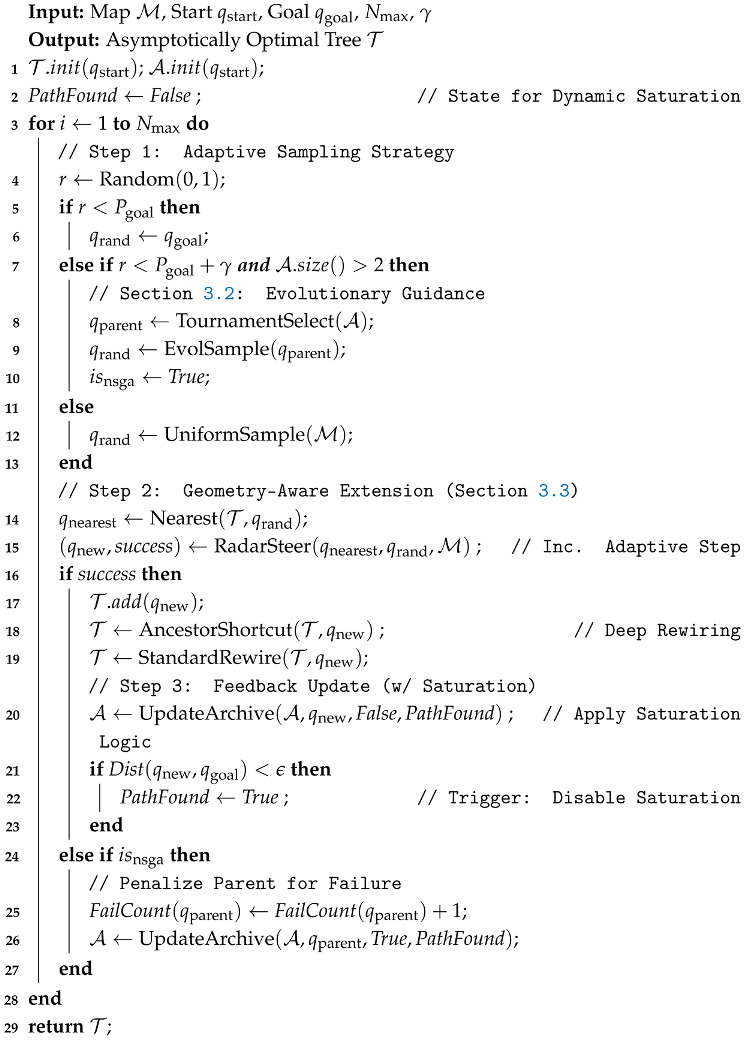


The seamless interaction between the two layers is achieved through a tightly coupled bidirectional feedback mechanism. In the top-down phase, the high-level NSGA-II module guides global exploration by selecting promising parent nodes from the Pareto front and assigning corresponding target directions. In the bottom-up phase, the geometry-aware execution layer carries out the local extension process. The core interface lies in dynamic status feedback. When a local extension fails; for example, due to a collision with the boundaries of a U-shaped trap during radar-guided steering, the failure is immediately reflected by increasing the parent node’s failure-count objective ($f_{2}$). The updated $f_{2}$ value is then returned to the high-level NSGA-II module for Pareto re-evaluation. Consequently, local geometric infeasibility can directly reshape the global search preference, guiding the planner to allocate resources toward safer and more viable topological branches. This mechanism ensures a coherent and robust hierarchical planning process.

### 3.2. Upper-Level Guidance: Multi-Objective Evolutionary Sampling

The probabilistic switch may activate the evolutionary branch, as described in the main workflow (Section 3.1). The sampling process departs from uniform random exploration and follows a guidance-driven strategy. This section details the internal mechanisms of the upper-level guidance. This guidance transforms the path planning problem into a dynamic multi-objective optimization task.

The search tree T is conceptualized as an evolving population, in which each node corresponds to an individual candidate solution. To effectively steer this population, a robust maintenance protocol is established for elite nodes, together with a generative strategy that takes advantage of the local kinematic inertia of the tree.

#### 3.2.1. Objective Space and Elite Maintenance

In order to distinguish between promising and stagnant nodes within the NSGA-II engine, each node $q_{i}\in V$ is mapped to a two-dimensional objective space:

(4) $$F\left(q_{i}\right)\;=\;{\left[f_{1}\left(q_{i}\right),\;f_{2}\left(q_{i}\right)\right]}^{T}.$$

- Convergence ($f_{1}$): The first objective is defined as the Euclidean distance to the goal, $d(q_{i},\;q_{\mathrm{goal}})$. This objective provides the greedy pressure required for asymptotic optimality.
- Viability ($f_{2}$): The second objective is defined as the cumulative failure count and serves as a collision penalty. Nodes that repeatedly lead to collisions during extension incur higher penalties, biasing the optimizer toward safer regions of the search space.

The dual objectives $f_{1}$ and $f_{2}$ are inherently conflicting in complex environments. A node located within a dead-end region may achieve a favorable convergence objective ($f_{1}$), while simultaneously incurring rapidly increasing collision penalties reflected by the failure-count objective ($f_{2}$). In contrast, a node located near the entrance of an unexplored narrow passage may exhibit a less favorable distance objective ($f_{1}$) while maintaining high feasibility, which is reflected by a low failure-count objective ($f_{2}$). Rather than combining these conflicting metrics into a static weighted sum, the NSGA-II engine maintains a dynamic balance through Pareto-based selection. This mechanism allows both types of nodes to be retained simultaneously in the elite archive, including those favoring aggressive convergence and those preserving exploratory safety. As a result, the algorithm reduces the risk of prematurely discarding safe nodes that may serve as critical topological bridges when greedy search paths become blocked.

To highlight the theoretical advantages of the proposed dual-objective formulation, it is instructive to compare the framework with recent hybrid approaches reported in the literature. Recent studies have integrated RRT with reinforcement learning (RL) to provide global guidance [20], or employed single-objective genetic algorithms (e.g., ERRT-GA) to optimize the initial sampling distribution [19]. Although these methods demonstrate improved convergence performance, they exhibit several structural limitations in practical warehouse logistics scenarios. RL-based approaches typically require extensive environment-specific pre-training and often lack generalization capability when applied to newly configured high-density layouts. In contrast, existing GA-based hybrid methods usually formulate path planning as a single-objective optimization problem (e.g., minimizing path length), which does not explicitly address the inherent trade-off between exploratory efficiency and navigational safety in narrow passages. By incorporating NSGA-II, the proposed framework provides a fundamentally different paradigm. It jointly evaluates the “distance to goal” ($f_{1}$) and “failure count” ($f_{2}$) without relying on pre-training, thereby enabling real-time Pareto-optimal trade-offs between exploration efficiency and geometric safety.

An elite archive A is maintained to store the Pareto-optimal frontier induced by the search tree. Formally, a node $q_{i}$ is defined to dominate another node $q_{j}$, denoted by $q_{i}≺q_{j}$, if and only if

(5) $$\forall k\;\in \;\left\{1,\;2\right\},\;f_{k}\left(q_{i}\right)\;\leq \;f_{k}\left(q_{j}\right)\;\wedge \;\exists k\;\in \;\left\{1,\;2\right\},\;f_{k}\left(q_{i}\right)\;<\;f_{k}\left(q_{j}\right).$$

At each iteration, the archive is updated by using the fast non-dominated sorting procedure, which stratifies the nodes into a sequence of non-dominated fronts $F_{1},\;F_{2},\;\ldots$, with $F_{1}$ representing the set of solutions achieving the best current trade-offs.

To mitigate the genetic drift [26] that may cause the population to cluster around a single local optimum, the crowding distance $\rho (q_{i})$ is calculated for each node. It is defined as the normalized sum of distances to the nearest neighboring nodes along each objective dimension:

(6) $$\rho \left(q_{i}\right)\;=\;\sum_{k=1}^{2}\frac{f_{k}\left(q_{i+1}\right)\;-\;f_{k}\left(q_{i-1}\right)}{f_{k,\max}\;-\;f_{k,\min}},$$

where $q_{i-1}$ and $q_{i+1}$ denote the adjacent nodes in the front sorted according to the *k*-th objective. To ensure computational tractability, the size of the archive is capped at $N_{\max}$. When the size of A exceeds the prescribed limit, a truncation procedure is applied. The nodes are retained in order of increasing dominance rank; within the rank that exceeds the capacity, the nodes with larger crowding distances are preferentially retained. This procedure eliminates redundant solutions while preserving diversity in the search tree.

#### 3.2.2. Inertia-Biased Ellipsoidal Sampling

Standard evolutionary algorithms commonly rely on isotropic Gaussian mutation, where samples are generated uniformly in all directions around a parent state. In narrow corridors, the valid configuration space is highly anisotropic. It often resembles a thin manifold aligned with the corridor axis. As a result, isotropic sampling exhibits a high rejection rate, since most generated samples fall within surrounding obstacles.

**Figure 1:**
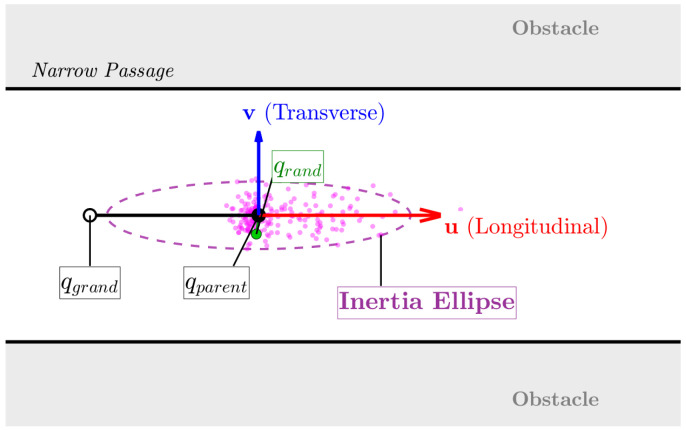
Schematic diagram of inertia-biased ellipsoidal sampling.

To address this geometric mismatch, an inertia-biased ellipsoidal sampling strategy is proposed. This method takes advantage of the growth history of the tree to capture the local structure of the free space, and is then used to derive an anisotropic sampling distribution. As shown in Figure 1, the principle of the inertia-biased ellipsoidal sampling strategy is illustrated.

Let $q_{\mathrm{parent}}$ be a node selected from A. When a parent node $q_{\mathrm{parent}}$ admits an ancestor $q_{\mathrm{grand}}$, the vector connecting these two nodes provides a local approximation of the tangent of the path and defines the associated kinematic inertia. A local coordinate frame is constructed, consisting of a longitudinal unit vector u and a transverse unit vector v:

(7) $$u\;=\;\frac{q_{\mathrm{parent}}\;-\;q_{\mathrm{grand}}}{\left|\right|q_{\mathrm{parent}}\;-\;q_{\mathrm{grand}}\left|\right|},\;v\;=\;u^{⊥}.$$

The candidate sample $q_{\mathrm{rand}}$ is generated by projecting independent Gaussian perturbations onto the local coordinate frame:

(8) $$q_{\mathrm{rand}}\;=\;q_{\mathrm{parent}}\;+\;\eta_{u}u\;+\;\eta_{v}v,$$

where $\eta_{u}∼N\left(0,\;\sigma_{\mathrm{long}}\cdot s_{k}\right)$ and $\eta_{v}∼N\left(0,\;\sigma_{\mathrm{trans}}\cdot s_{k}\right)$. By selecting $\sigma_{\mathrm{long}}\;\gg \;\sigma_{\mathrm{trans}}$ (e.g., with a ratio of 12:1), the resulting probability density function assumes an ellipsoidal shape elongated along the direction of motion, thus increasing the likelihood of successfully traversing narrow gaps. To discourage backtracking, the magnitude of the longitudinal component $\eta_{u}$ is dampened (e.g., scaled by a factor of 0.5) whenever it takes a negative value. In addition, a dynamic scaling factor $s_{k}$ is introduced to implement a multi-resolution search strategy. Within a single sampling burst of $K_{\mathrm{burst}}$ attempts, $s_{k}$ decays in a stepwise manner (e.g., from 1.0 to 0.25) as the attempt index *k* increases. This strategy follows a “coarse-to-fine” paradigm: the algorithm first attempts larger exploratory steps to traverse open regions efficiently; when such attempts fail, the sampling variance is progressively reduced to enable fine-grained adjustments required to negotiate extremely narrow bottlenecks. The integrated procedure for parent selection and inertia-based sampling is detailed in Algorithm 2.

**Algorithm 2:** Multi-Objective Evolutionary Sampling Strategy

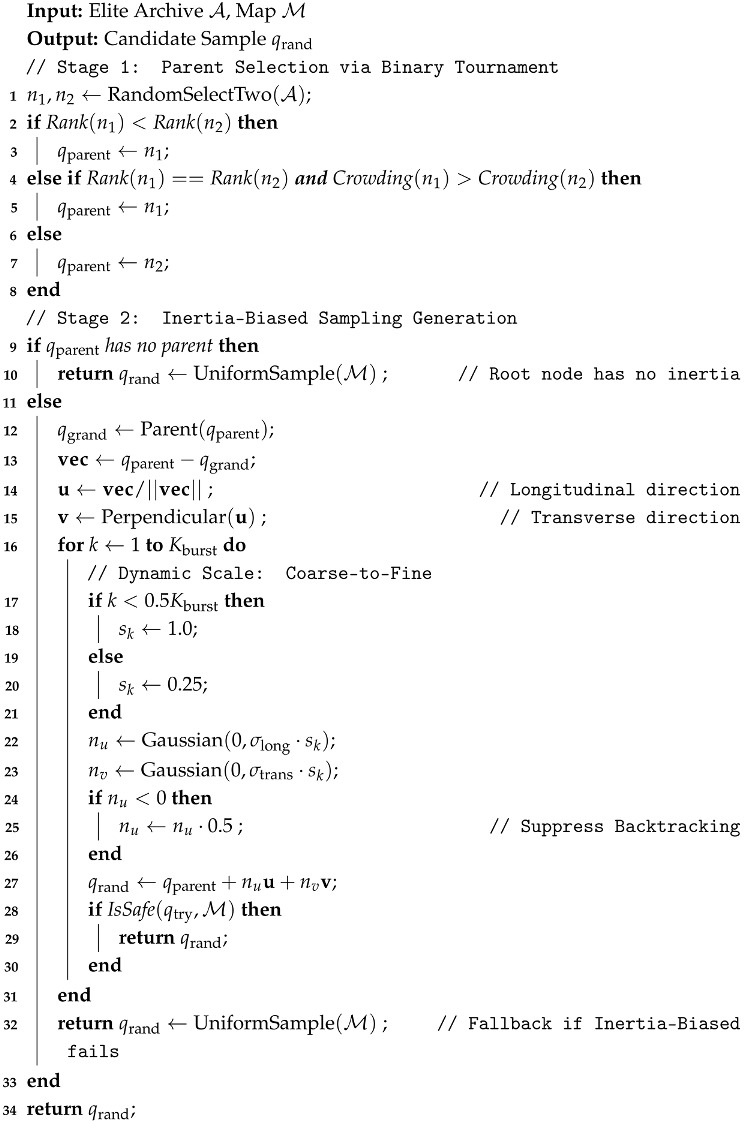



### 3.3. Lower-Level Execution: Geometry-Aware Local Extension

While where to explore is determined by upper-level guidance, how to traverse the identified regions is governed by the lower-level execution layer. The standard RRT* expansion relies on a fixed step size and direct line-of-sight connections. This strategy is particularly fragile in narrow passages due to the severely constrained valid configuration space. To mitigate these geometric deficiencies, a suite of geometry-aware mechanisms is introduced. These mechanisms aim to improve local extension efficiency and refine the resulting path topology.

#### 3.3.1. Radar-Guided Steering with Adaptive Step-Size

**Figure 2:**
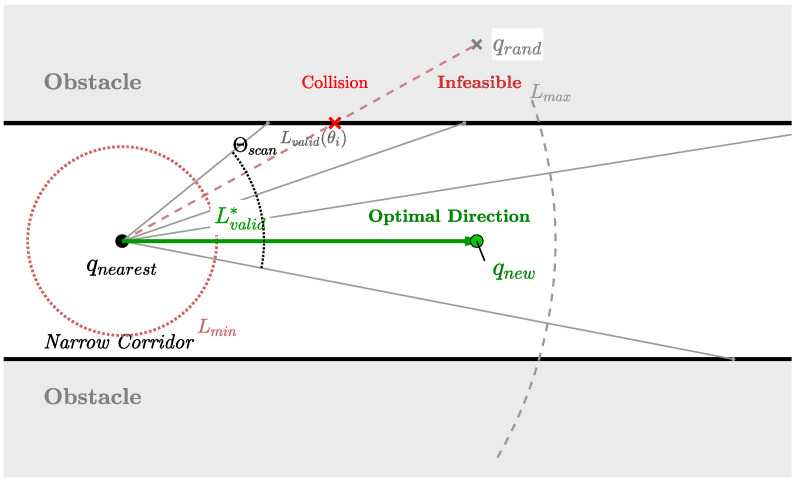
Schematic diagram of radar-guided steering.

In constrained environments, the direction vector from the nearest node $q_{\mathrm{nearest}}$ toward the sampled target $q_{\mathrm{rand}}$ frequently collides with obstacles, leading to failed extensions. A radar-guided steering strategy is proposed to alleviate this issue, which emulates a local sensory scanning process. The specific principle is illustrated in Figure 2.

Rather than strictly following the direction ${\vec{v}}_{\mathrm{target}}\;=\;q_{\mathrm{rand}}\;-\;q_{\mathrm{nearest}}$, a discrete set of angular deviations $\Theta_{\mathrm{scan}}\;=\;\left\{0,\;\pm \phi_{1},\;\pm \phi_{2},\;\ldots ,\;\pm \phi_{k}\right\}$ is systematically explored around ${\vec{v}}_{\mathrm{target}}$. For each candidate angle $\theta \in \Theta_{\mathrm{scan}}$, an adaptive step-size procedure is applied to determine the maximum feasible extension length.

Let $L_{\max}$ be the nominal maximum step size. The adaptive procedure is used to compute the maximum collision-free distance $L_{\mathrm{valid}}\left(\theta \right)$ along the direction θ by using a binary search method:

(9) $$L_{\mathrm{valid}}\left(\theta \right)\;=\;\sup\left\{l\;\in \;\left[0,\;L_{\max}\right]|\mathrm{CollisionFree}\left(q_{\mathrm{nearest}},\;q_{\mathrm{nearest}}\;+\;l\cdot {\vec{u}}_{\theta }\right)\right\}.$$

where ${\vec{u}}_{\theta }$ is the unit vector derived from rotating ${\vec{v}}_{\mathrm{target}}$ by θ. The algorithm selects the optimal extension $\left(L^{*},\;\theta^{*}\right)$ that maximizes the extension distance:

(10) $$\left(L^{*},\;\theta^{*}\right)\;=\;\underset{\theta \in \Theta_{\mathrm{scan}}}{\arg\max}L_{\mathrm{valid}}\left(\theta \right).$$

This collision-aware binary search mechanism inherently regulates the spatial sampling resolution in accordance with local geometric constraints. In open and obstacle-free regions, the algorithm consistently employs the maximum allowable step size (MaxStep), resulting in sparse node distribution and efficient traversal, i.e., a low sampling resolution. In contrast, when navigating through narrow passages or in close proximity to obstacle boundaries, the binary search procedure is invoked to iteratively reduce the step size until the maximum collision-free distance ($L_{valid}<MaxStep$) is obtained. This adaptive adjustment locally increases the sampling resolution, producing shorter and denser expansions. Such a mechanism ensures the geometric precision required for safe navigation in constrained environments, while maintaining high expansion efficiency in free space, thereby eliminating the need for manual tuning of the sampling resolution.

Subject to a minimum effectiveness threshold $L_{\min}$, this mechanism promotes tree expansion along the longitudinal direction of narrow corridors. In complex U-shaped traps, where the direct line of sight is severely obstructed, the distance-maximization strategy naturally identifies oblique directions that are approximately parallel to the trap boundaries. Consequently, the algorithm tends to advance along the obstacle boundaries to locate the exit, rather than repeatedly extending into the dead-end region. As a result, tree growth is guided along obstacle boundaries, significantly reducing the likelihood of stagnation in highly constrained regions.

#### 3.3.2. Topology Refinement via Ancestor Shortcut

**Figure 3:**
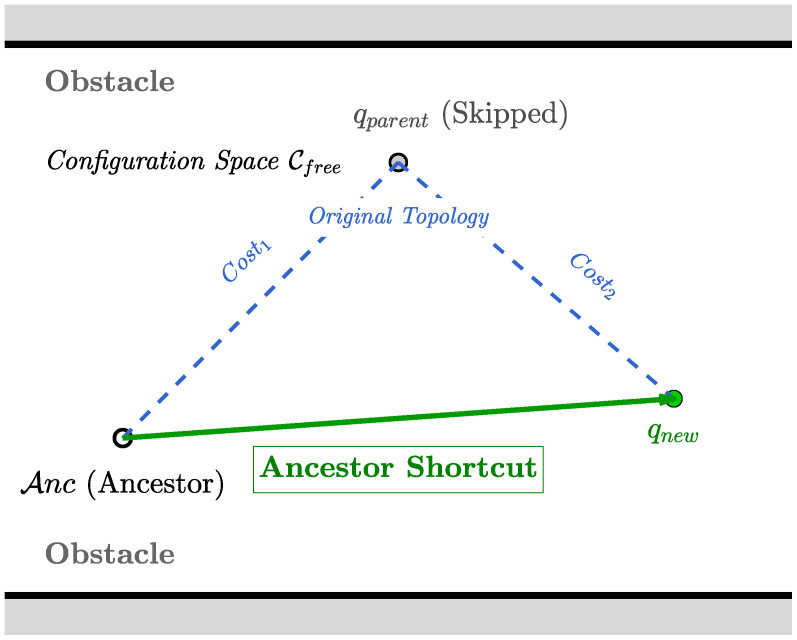
Schematic diagram of ancestor shortcut.

To limit computational complexity, RRT* typically employs a small rewiring radius. In narrow passages, this restriction often leads to paths with jagged and oscillatory structures. This zig-zag behavior degrades the quality of the path and hampers local refinement. A deep rewiring strategy termed ancestor shortcut is introduced to alleviate this issue. The specific principle is illustrated in Figure 3.

After a new node $q_{\mathrm{new}}$ is successfully added to the tree, a direct connection to higher-order ancestors is evaluated. Specifically, the algorithm considers bypassing the immediate parent by attempting a shortcut to an ancestor node, such as the grandparent $q_{\mathrm{grand}}\;=\;\mathrm{Parent}\left(q_{\mathrm{parent}}\right)$. Let $\mathrm{Anc}(q_{\mathrm{new}},\;d)$ denote the ancestor of $q_{\mathrm{new}}$ at depth *d*. A shortcut connection is considered valid if the following conditions are met:

(11) $$\mathrm{CollisionFree}\left(Anc\left(q_{\mathrm{new}},\;d\right),\;q_{\mathrm{new}}\right)\wedge \mathrm{Cos}t\left(Anc\right)\;+\;\left|\right|q_{\mathrm{new}}\;-\;Anc\left|\right|\;<\;\mathrm{Cos}t\left(q_{\mathrm{new}}\right).$$

here, CollisionFree(u, v) denotes a boolean function that is evaluated to true if the straight-line segment connecting nodes *u* and *v* lies entirely within the collision-free space $C_{\mathrm{free}}$. Cost(u) denotes the accumulated path cost from the root node $q_{\mathrm{start}}$ to the node *u* along the tree structure, measured as the sum of Euclidean distances. The operator ||·|| denotes the Euclidean norm between configurations. This condition guarantees that the proposed shortcut is geometrically feasible and yields a strict reduction in the total length of the path. As a result, the local topology of the search tree is refined, enabling path smoothing and improved cost convergence without requiring a larger neighbor search radius.

This dynamic rewiring process provides two key practical benefits. First, it directly reduces the overall path length. By bypassing intermediate nodes and establishing direct line-of-sight connections with higher-level ancestors, the algorithm exploits the triangle inequality to eliminate unnecessary detours. Second, it improves the kinematic feasibility of the resulting trajectory for AGVs. Standard random sampling in constrained environments often produces oscillatory (zig-zag) path segments, which require frequent deceleration and sharp steering adjustments. The ancestor shortcut mechanism functions as a real-time topological smoothing strategy by directly connecting distant nodes and removing redundant intermediate points. This refinement transforms irregular connections into longer and smoother segments, thereby reducing steering effort and enabling a more continuous velocity profile during execution.

#### 3.3.3. Failure Feedback Mechanism

To close the loop between local execution and global guidance, a failure feedback mechanism is incorporated. If the Radar-Guided Steering procedure fails to identify any valid extension (i.e., $\forall \theta ,\;L_{\mathrm{valid}}\left(\theta \right)\;<\;L_{\min}$), the expansion attempt is classified as failure.

Specifically, when a failed extension originates from an evolutionary sample (indicated by the flag $is_{\mathrm{nsga}}$), the failure count objective $f_{2}$ associated with the parent node $q_{\mathrm{parent}}$ is incremented:

(12) $$f_{2}\left(q_{\mathrm{parent}}\right)\leftarrow f_{2}\left(q_{\mathrm{parent}}\right)\;+\;1.$$

This penalty degrades the dominance rank of $q_{\mathrm{parent}}$ in subsequent NSGA-II sorting. Therefore, the likelihood of selecting nodes trapped in dead-end regions is adaptively reduced, enabling computational resources to be reallocated toward unexplored and geometrically feasible frontiers. The geometry-aware extension procedure is detailed in Algorithm 3.
**Algorithm 3:** Geometry-Aware Local Extension Algorithm
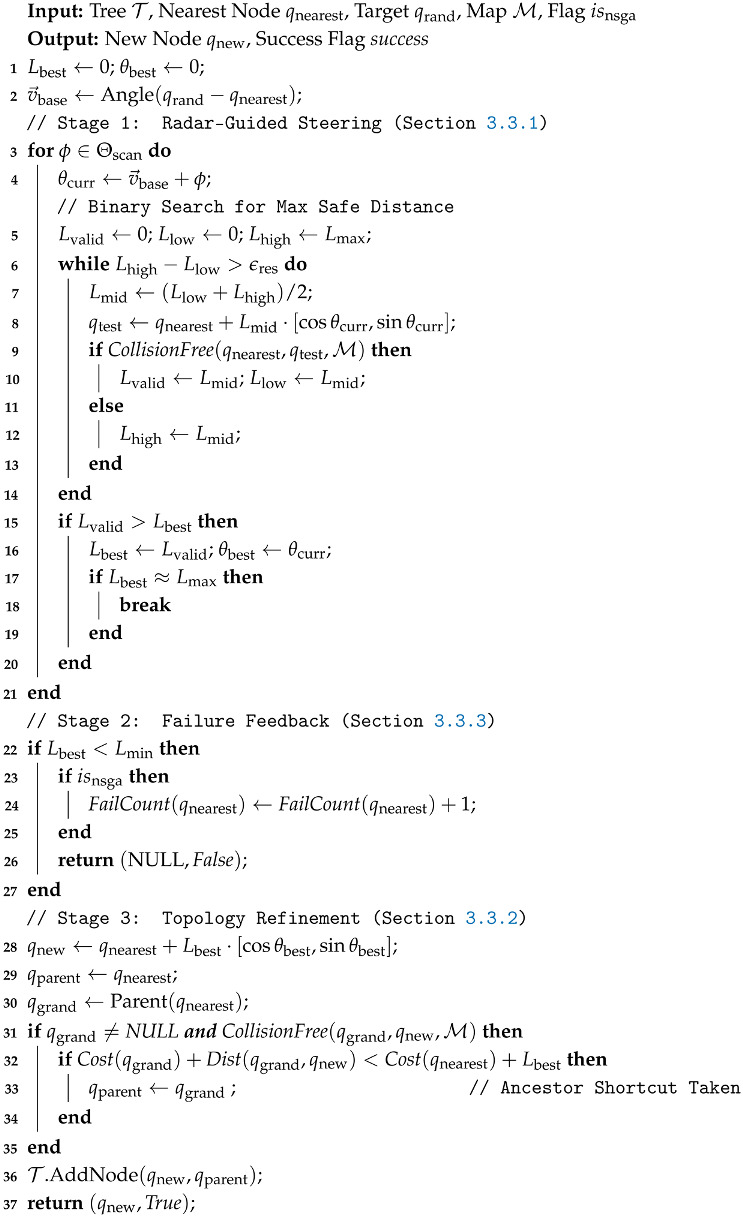


## 4. Experiments and Analysis

In this section, a series of experiments are performed to evaluate the performance of the proposed MEG-RRT* algorithm. The experimental evaluation is conducted with two primary objectives: (1) to demonstrate that the proposed multi-objective evolutionary guidance improves the success rate and convergence behavior in geometrically constrained environments compared with state-of-the-art sampling-based planners; (2) to evaluate the ability of the geometry-aware execution mechanisms to generate smooth and high-quality paths suitable for AGV operation in realistic warehouse scenarios.

### 4.1. Experiment Settings

#### 4.1.1. Simulation Environment

All simulations were implemented in MATLAB R2025a, and the hardware environment consisted of a workstation equipped with an Intel Core i7-14700K CPU @ 3.40 GHz and 64 GB of RAM (Acer Inc., New Taipei City, China). To ensure statistical reliability, each algorithm was executed 100 times for each scenario, with the maximum number of iterations set to $N_{\max}\;=$ 10,000. To ensure that the planned trajectories are both strictly collision-free and practically feasible for physical deployment, a uniform safety margin (SafetyMargin = 0.1 m) is incorporated into the continuous collision detection module. This margin effectively inflates obstacle boundaries to account for the physical footprint of the AGVs, ensuring minimum clearance during radar-guided steering, local rewiring, and ancestor shortcut operations in high-density warehouse environments.

**Figure 4:**
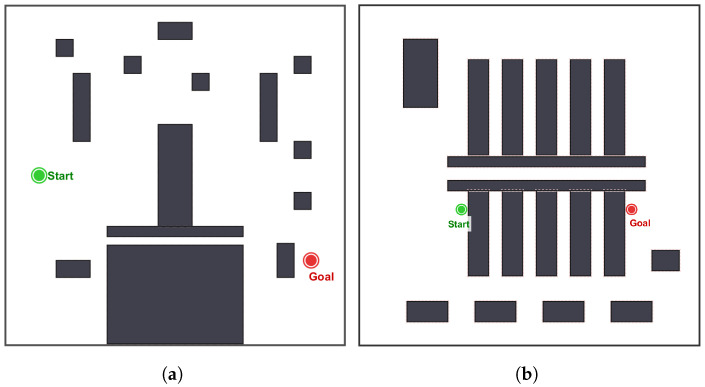
Two distinct simulation scenarios: (**a**) The complex narrow passage scenario. (**b**) The warehouse logistics simulation scenario.

Two distinct simulation scenarios are designed to evaluate the algorithm from multiple perspectives:

- Scenario A: complex narrow passage, as shown in Figure 4a. A theoretically challenging environment is constructed, featuring U-shaped traps and narrow corridors. This map is intended to evaluate the algorithm’s capability to escape local minima and traverse low-connectivity bottlenecks.
- Scenario B: warehouse logistics environment, as shown in Figure 4b. A high-density warehouse environment is simulated, characterized by long, parallel aisles and densely arranged shelving units. This scenario is designed to evaluate the practical applicability of the algorithm to AGV navigation, with particular emphasis on path smoothness and alignment with corridor axes.

#### 4.1.2. Comparison Algorithms and Parameter Settings

The proposed MEG-RRT* is compared with three representative sampling-based algorithms:

- RRT*: The standard asymptotically optimal planner with uniform sampling.
- Informed-RRT*: A variant that constrains sampling within a heuristic ellipsoid to accelerate convergence.
- RRT-Connect: A bi-directional extension strategy characterized by high search efficiency, though not asymptotically optimal.

The specific parameters of the four algorithms are listed in Table 1.

#### 4.1.3. Performance Metrics

To ensure statistical reliability for sampling-based planners with inherent probabilistic behavior, the quantitative evaluation is conducted based on 100 independent Monte Carlo trials for each scenario. The performance of each method is assessed using the following five metrics:

- Success Rate (SR): The percentage of trials in which a collision-free path connecting the start and goal regions is successfully found within the prescribed limit of $N_{\max}$ iterations.
- Iterations to First, Solution ($I_{\mathrm{first}}$): The number of iterations required to obtain the first feasible path. This metric provides a hardware-independent measure of the exploratory efficiency of the algorithm.
- Computation Time ($T_{\mathrm{exec}}$): The average CPU time, measured in seconds, required to obtain the first feasible solution. This metric directly reflects the actual computational cost, including the overhead associated with collision checking, nearest-neighbor search, and evolutionary operations.
- Final Path Cost ($C_{\mathrm{best}}$): The Euclidean length of the best path obtained after the maximum number of iterations, $N_{\max}$. This metric reflects the asymptotic optimality and kinematic quality of the final solution.
- Tree Size/Memory Efficiency ($N_{\mathrm{nodes}}$): This metric denotes the total number of valid configurations (nodes) maintained in the search tree when the first feasible solution is found. A lower value indicates a smaller memory footprint and reflects the algorithm’s ability to avoid redundant sampling in obstructed regions.

### 4.2. Experiment and Analysis

#### 4.2.1. Scenario A: Performance in Complex Narrow Passages

This scenario serves as a theoretical benchmark to reveal the limitations of sampling-based planners in geometrically constrained environments. The map consists of rectangular obstacles with uneven spatial distribution and varying sizes. This configuration facilitates the evaluation of the algorithm’s ability to escape local minima and traverse narrow passages.

Table 2 summarizes the statistical performance of the four algorithms in 100 independent trials in scenario A. Under the iteration limit of $N_{\max}\;=$ 10,000, all algorithms achieved a success rate of 100%. Nevertheless, noticeable differences remain in terms of convergence behavior and solution quality.

Exploration Efficiency ($I_{\mathrm{first}}$): The MEG-RRT* exhibits superior efficiency in identifying the initial solution, achieving a mean iteration count of 299. This represents a 43.5% reduction compared with standard RRT* (529) and a 44.9% reduction compared with Informed-RRT* (543). Although RRT-Connect achieves a slightly lower mean iteration count (262), this advantage is obtained at the expense of asymptotic optimality, as discussed later. In particular, the standard deviation of MEG-RRT* (±136.9) is significantly lower than that of Informed-RRT* (±231.2), indicating that our method offers superior stability and is less prone to extreme outliers caused by random sampling failures in narrow gaps. This efficiency can be attributed to the radar-guided steering, which aligns tree growth with the corridor axis and reduces wasted samples on obstacle surfaces.

Computation Time ($T_{\mathrm{exec}}$): In terms of absolute execution time, standard RRT*, Informed-RRT*, and RRT-Connect achieve extremely low mean computation times of 0.0031 s, 0.0032 s, and 0.0011 s, respectively. By contrast, the proposed MEG-RRT* requires an average of 0.2503 s to obtain the first feasible path. This increase in CPU time is an expected and deliberate trade-off. The baseline algorithms rely on computationally inexpensive uniform sampling and simple line-of-sight steering, which are fast in each iteration but often suffer from severe stagnation and unguided exploration in U-shaped traps. In contrast, MEG-RRT* introduces greater computational complexity per iteration through NSGA-II-based elite archive sorting, crowding-distance calculation, and multi-angle radar-guided steering. Although these geometry-aware and evolutionary operations impose additional computational overhead, they substantially reduce aimless trial-and-error during exploration. It should also be noted that an initial global planning time of approximately 0.25 s remains highly efficient and is fully compatible with the operational requirements of practical AGV dispatch systems, especially considering the significant improvements in path quality and exploration stability.

Solution Optimality ($C_{\mathrm{best}}$): In terms of path quality, MEG-RRT* achieves the lowest mean path cost (92.8), outperforming Informed-RRT* that focused on optimization (93.3). This marginal yet consistent superiority validates the effectiveness of the ancestor shortcut mechanism, which aggressively straightens the path topology during the exploration phase. However, standard rewiring (limited by the small radius r = 5.0) requires significantly more iterations to smooth out the “zig-zag” artifacts. In contrast, RRT-Connect generates substantially longer paths (149.5) despite its high search speed, indicating a pronounced trade-off between efficiency and solution quality. Such path characteristics limit its applicability to energy-efficient AGV operations.

Tree size ($N_{nodes}$): RRT-Connect employs the smallest number of nodes (175), which is attributable to its greedy bi-directional expansion strategy. In MEG-RRT*, Samples are preferentially allocated to critical regions under NSGA-II guidance, which facilitates bottleneck traversal while maintaining a moderate memory footprint and high solution quality. It should be noted that MEG-RRT* exhibits a relatively large standard deviation in tree size (±162.0). This variation is a natural consequence of the adaptive evolutionary operators and the dynamic failure-feedback mechanism. In trials where the initial elite nodes are quickly aligned with the entrance of a narrow corridor, the target can be reached with a relatively small tree. In contrast, when the initial exploration penetrates deeply into U-shaped traps, the algorithm must expand more nodes to accumulate failure penalties, which in turn drives the search to escape local minima. This structural flexibility enables the algorithm to handle severe geometric traps effectively. More importantly, despite this variation in memory footprint, the final path cost of MEG-RRT* remains highly stable, with a standard deviation of only ±5.2. This result indicates that the proposed method can consistently converge to high-quality solutions under different initial topological conditions.

**Figure 5:**
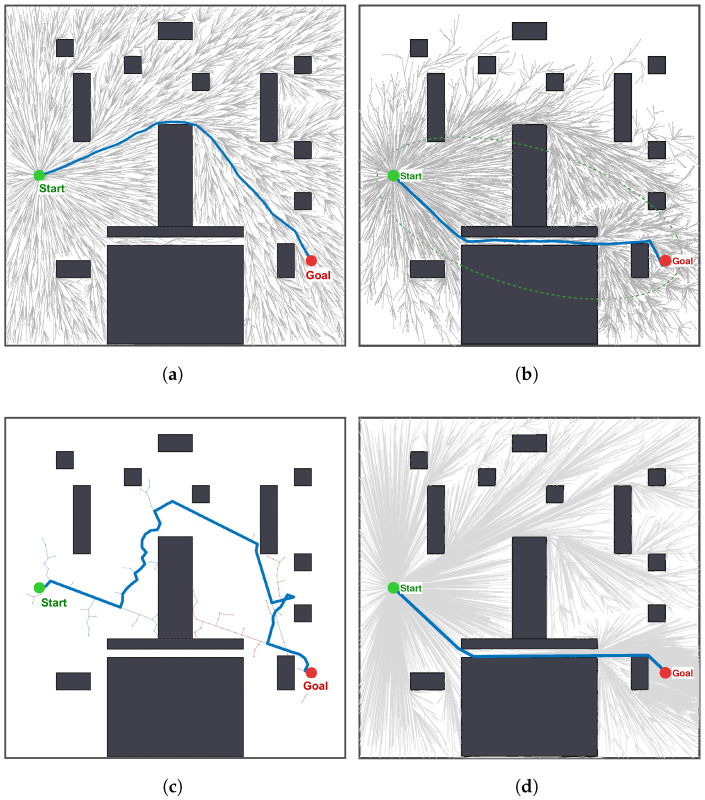
Path planning results of four algorithms in scenarios A: (**a**) RRT* ($I_{\mathrm{first}}\;=\;495$, $C_{\mathrm{best}}\;=\;103.4$, Nodes for Feasible Sol. = 342). (**b**) Informed-RRT* ($I_{\mathrm{first}}\;=\;910$, $C_{\mathrm{best}}\;=\;93.5$, Nodes for Feasible Sol. = 494). (**c**) RRT-Connect ($I_{\mathrm{first}}\;=\;218$, $C_{\mathrm{best}}\;=\;156.7$, Nodes for Feasible Sol. = 183). (**d**) MEG-RRT* ($I_{\mathrm{first}}\;=\;161$, $C_{\mathrm{best}}\;=\;89.8$, Nodes for Feasible Sol. = 221).

As illustrated in Figure 5, the observed quantitative differences are visually explained by the topological differences among the generated paths. A characteristic exploration pattern can be observed in standard RRT* (Figure 5a). The search tree densely populates the open region of the U-shaped trap but shows limited progress through the narrow exit. This behavior indicates that RRT* converges to a local minimum. As shown in Figure 5b, Informed-RRT* improves upon Standard RRT* by restricting sampling to a heuristic ellipsoidal region. This strategy yields a cleaner tree structure and a more optimized path than RRT*. However, in non-convex environments such as a U-shaped trap, the ellipsoidal region still encompasses a substantial portion of obstructed space. Redundant sampling around obstacles is still present, though at a reduced level. RRT-Connect (Figure 5c) finds a solution quickly but produces a highly erratic trajectory that closely follows obstacle boundaries. This behavior is a direct consequence of its greedy connection strategy and is kinematically unfavorable for AGV operation.

In contrast, MEG-RRT* (Figure 5d) generates a smooth and direct path that traverses the narrow corridor with minimal oscillation. The corresponding node distribution indicates a structured sampling behavior, with samples concentrated along the axis of the critical bottleneck rather than dispersed in open dead-end regions. These observations demonstrate the effectiveness of the Radar-Guided Steering in aligning tree growth with narrow passages, as well as the role of the Ancestor Shortcut in straightening the local pathtopology. Moreover, the extended step lengths observed along the trajectory highlight the contribution of the Adaptive Step-size mechanism to efficient expansion in free space.

The comparative results in Scenario A indicate that the MEG-RRT* effectively mitigates the narrow passage problem. The method attains a favorable trade-off between convergence efficiency and solution optimality by combining evolutionary guidance with geometry-aware execution. This integrated design avoids both the premature stagnation commonly observed in optimization-driven planners and the jagged path characteristics produced by greedy strategies.

#### 4.2.2. Scenario B: Performance in Warehouse Logistics Environment

This scenario represents a high-density warehouse environment characterized by long, parallel aisles and densely arranged shelving units. Unlike Scenario A, which emphasizes topological complexity, this environment places greater emphasis on the smoothness of the path and the alignment with the corridor axes. These properties are essential to ensure the kinematic feasibility and energy efficiency in AGV operation.

Table 3 summarizes the statistical performance of the four algorithms in 100 independent trials in scenario B. Under the iteration limit of $N_{\max}=$ 10,000, all algorithms achieved a success rate of 100%. Nevertheless, noticeable differences remain in terms of convergence behavior and solution quality.

Exploration Efficiency ($I_{\mathrm{first}}$): RRT-Connect exhibits the fastest exploration, with a mean $I_{\mathrm{first}}=389$. This performance is attributed to its greedy bi-directional extension strategy, which rapidly connects the start and goal trees in open aisles. MEG-RRT* demonstrates competitive exploration efficiency, with a mean $I_{\mathrm{first}}=406$. Its performance is only slightly slower than that of RRT-Connect and substantially better than that of Standard RRT* (1069) and Informed-RRT* (994). The standard deviation of MEG-RRT* (±245.8) is substantially lower than that of RRT* (±490.4) and Informed-RRT* (±486.4). This result indicates that the radar-guided steering yields a more consistent search behavior along the aisles and reduces the variability introduced by random sampling in large-scale environments.

Computation Time ($T_{\mathrm{exec}}$): Consistent with the observations in Scenario A, the baseline algorithms achieve extremely short per-trial execution times because of their simple heuristic structures, resulting in near-instantaneous discovery of the first feasible solution (e.g., 0.0015 s for RRT-Connect and 0.0065 s for standard RRT*). By contrast, the proposed MEG-RRT* exhibits a higher mean execution time of 0.3377 s. In a large-scale warehouse environment with long aisles, the comprehensive geometric scanning and multi-objective sorting performed in each iteration naturally introduce a higher computational burden. However, this additional fraction of a second in the planning phase directly translates into a highly structured and kinematically smooth trajectory with the lowest overall path cost. For industrial AGVs, a 39% reduction in physical travel distance compared with RRT-Connect, together with the avoidance of erratic sharp turns, can yield operational time and energy savings that outweigh the additional 0.33 s computational overhead. Therefore, the proposed framework maintains a practical balance between algorithmic planning time and physical execution efficiency.

Solution Optimality ($C_{\mathrm{best}}$): MEG-RRT* achieves the best path quality with a mean cost of 81.5, outperforming the optimization-centric Informed-RRT* (82.1). The lower standard deviation (±2.3) further confirms its robustness. This improvement is attributed to the ancestor shortcut mechanism, which effectively eliminates redundant waypoints and straightens the path along the long corridors. In contrast, a substantially higher path cost (113.6) is generated by RRT-Connect, which is approximately 39% longer than that of MEG-RRT*. These characteristics are unfavorable for efficient AGV operation.

Tree size ($N_{nodes}$): Although RRT-Connect uses the fewest nodes (142), the quality of the resulting path is compromised. MEG-RRT* maintains a node count (512) comparable to Informed-RRT* (520), but achieves a better solution. These results indicate that the proposed method allocates computational resources more effectively. Sampling efforts are focused on critical topological routes rather than dispersed across the entire free space, enabling the planner to produce paths that are both feasible and near-optimal for logistical applications.

**Figure 6:**
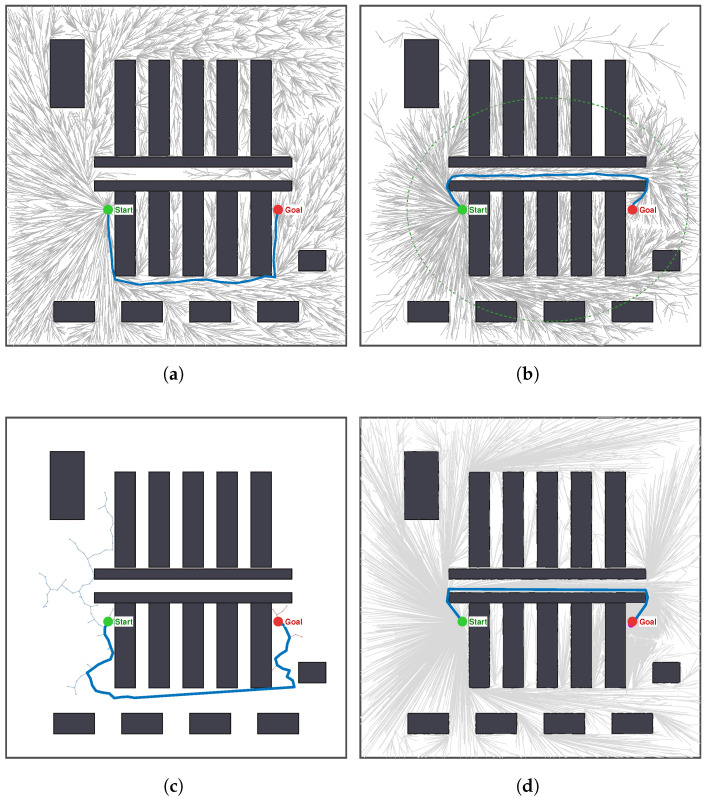
Path planning results of four algorithms in scenarios B: (**a**) RRT* ($I_{\mathrm{first}}\;=\;1040$, $C_{\mathrm{best}}\;=\;88.9$, Nodes for Feasible Sol. = 553). (**b**) Informed-RRT* ($I_{\mathrm{first}}\;=\;1644$, $C_{\mathrm{best}}\;=\;82.6$, Nodes for Feasible Sol. = 793). (**c**) RRT-Connect ($I_{\mathrm{first}}\;=\;336$, $C_{\mathrm{best}}\;=\;111.1$, Nodes for Feasible Sol. = 125). (**d**) MEG-RRT* ($I_{\mathrm{first}}\;=\;675$, $C_{\mathrm{best}}\;=\;79.9$, Nodes for Feasible Sol. = 873).

The visual comparisons presented in Figure 6 further corroborate the quantitative results. The standard RRT* (Figure 6a) shows a broad exploration pattern. The search tree densely expands in open regions but makes limited progress through narrow bottlenecks; this behavior indicates convergence to a local minimum. Informed-RRT* (Figure 6b) restricts sampling to a heuristic ellipsoidal region; this leads to a cleaner tree structure and a more optimized path. In non-convex environments, the ellipsoidal region still covers a substantial amount of obstructed space, the sampling efficiency remains limited. As a result, the sampling efficiency remains limited. RRT-Connect (Figure 6c) establishes connectivity rapidly, but generates trajectories that closely follow obstacle boundaries and contain sharp turns. These kinematic behaviors increase both the risk of collisions and the energy consumption of AGVs.

MEG-RRT* (Figure 6d) generates a smooth, centerline-aligned path through the long corridor. Nodes are sparsely distributed in non-critical regions and structured along the optimal route. This behavior demonstrates the effectiveness of radar-guided steering in maintaining corridor alignment and of the ancestor shortcut mechanism in removing high-frequency geometric artifacts along the path. In particular, the extended step lengths observed in the trajectory indicate the active contribution of the adaptive step-size mechanism to efficient expansion in free space.

In summary, a comprehensive comparison of execution time, memory usage, and path cost highlights the distinct operational characteristics of each planner. Although RRT-Connect demonstrates minimal memory usage and rapid initial connectivity, its greedy strategy often produces tortuous and suboptimal paths. Therefore, it is less suitable for AGV logistics, where kinematic smoothness and energy efficiency are critical. In contrast, conventional sampling-based methods, such as RRT* and Informed-RRT*, struggle to handle the geometric bottlenecks of warehouse aisles, resulting in larger memory footprints without consistently achieving superior path quality. The proposed MEG-RRT* intentionally accepts a modest increase in computational planning time (e.g., approximately 0.33 s per query) in exchange for a highly structured and geometry-aware search process. This trade-off allows the method to maintain a moderate memory footprint ($N_{\mathrm{nodes}}\;=\;512$) while reducing the physical path cost by approximately 39% compared with RRT-Connect, thus demonstrating its superior suitability and overall effectiveness for practical automated warehouse operations.

## 5. Conclusions

This paper presents MEG-RRT*, a novel path planning framework designed for AGVs operating in constrained warehouse environments. The proposed approach overcomes the limitations of uniform sampling in narrow passages. It combines global evolutionary guidance with local geometry-aware execution. The core contributions are threefold. First, NSGA-II is integrated to maintain a diverse and high-quality set of parent nodes at the exploration frontier. Second, a radar-guided steering strategy is developed to improve extension efficiency in constrained regions. Third, an ancestor shortcut mechanism is introduced to improve path smoothness through topology refinement.

Extensive benchmark experiments verify the effectiveness of the proposed method with clear quantitative evidence. In geometrically complex narrow-passage scenarios, MEG-RRT* achieves a 100% success rate and reduces the iterations required to obtain the first feasible solution by 43.5% and 44.9% compared with standard RRT* and Informed-RRT*, respectively. Furthermore, in high-density warehouse environments, the proposed method effectively suppresses jagged trajectories and generates kinematically smooth paths that are approximately 39% shorter than those produced by the greedy RRT-Connect algorithm. Notably, MEG-RRT* achieves these high-quality paths while maintaining a practical computation time of less than 0.35 s per query. These results demonstrate that MEG-RRT* effectively balances rapid exploration with high path quality, making it well suited for industrial AGV applications.

Despite these demonstrated advantages, the proposed framework still exhibits certain limitations. First, the integration of the NSGA-II optimization module and comprehensive geometric evaluation introduces a moderate computational overhead. Although the average planning time (approximately 0.35 s) is acceptable for static or quasi-static warehouse scenarios, it may limit real-time responsiveness in highly dynamic environments. Second, the current approach primarily operates as a geometric path planner. Strict nonholonomic kinodynamic constraints (e.g., velocity and acceleration limits) are handled implicitly through trajectory smoothing rather than being explicitly incorporated into the state-space sampling process.

To address these limitations and further enhance the practical applicability of the proposed framework, future research will focus on two primary directions. First, the planner will be extended to dynamic environments by incorporating real-time replanning mechanisms and advanced initialization strategies, such as warm-start methods, to effectively handle moving obstacles, including human workers and forklifts. Second, inspired by the effectiveness of evolutionary algorithms in complex logistics, future work will investigate multi-AGV collaborative planning. The population-based characteristics of the NSGA-II engine will be leveraged to support multi-robot task allocation. This mechanism also enables implicit trajectory coordination, thereby mitigating traffic conflicts in high-density warehouse environments.

## Figures and Tables

**Table 1:** The specific parameters of the four algorithms.

Category	Parameter	Symbol	RRT*	Informed-RRT*	RRT-Connect	MEG-RRT*
Environment	Map Size	-	100×100	100×100	100×100	100×100
	Max Iterations	$N_{\max}$	10,000	10,000	10,000	10,000
	Safety Margin	δ	0.1	0.1	0.1	0.1
	Goal Threshold	ϵ	2.0	2.0	2.0	2.0
	Start/Goal	-	[10, 50]/[90, 25]			
RRT Basics	Step Size	η	2.0	2.0	2.0	0.2–5.0 (Adaptive)
	Rewire Radius	*r*	5.0	5.0	N/A	5.0
	Goal Bias	$P_{\mathrm{goal}}$	0.05	0.05	N/A	0.05
	Bi-Directional	-	No	No	Yes	No
MEG-Specific	NSGA Sample Rate	γ	-	-	-	0.5
	Archive Size	|A|	-	-	-	100
	Burst Attempts	$K_{\mathrm{burst}}$	-	-	-	20
	Dist. Saturation	$\delta_{\mathrm{sat}}$	-	-	-	20.0
	Radar Scan	$\Theta_{\mathrm{scan}}$	-	-	-	$\pm 18^{\circ }$

**Table 2:** Comparison of performance metrics across four algorithms in Scenario A (100 trials).

Metric	RRT*	Informed-RRT*	RRT-Connect	MEG-RRT*
Success Rate (SR)	100%	100%	100%	100%
Iterations to First, Solution ($I_{\mathrm{first}}$)				
Mean ± Std	529 ± 184.2	543 ± 231.2	262 ± 115.9	299 ± 136.9
[Min, Max]	[249, 1231]	[246, 1544]	[73, 801]	[90, 715]
Computation Time ($T_{exec}$) [s]				
Mean ± Std	0.0031 ± 0.0015	0.0032 ± 0.0018	0.0011 ± 0.0011	0.2503 ± 0.1157
[Min, Max]	[0.0014, 0.0136]	[0.0013, 0.0148]	[0.0005, 0.0115]	[0.0466, 0.5630]
Final Path Cost ($C_{\mathrm{best}}$)				
Mean ± Std	100.5 ± 5.2	93.3 ± 6.3	149.5 ± 14.0	92.8 ± 5.2
[Min, Max]	[90.1, 105.4]	[89.4, 125.1]	[109.9, 189.5]	[89.1, 103.4]
Tree Size/Memory Efficiency ($N_{nodes}$)				
Mean ± Std	347 ± 108.1	351 ± 125.4	175 ± 46.3	385 ± 162.0
[Min, Max]	[181, 766]	[169, 909]	[85, 352]	[124, 864]

**Table 3:** Comparison of performance metrics across four algorithms in Scenario B (100 trials).

Metric	RRT*	Informed-RRT*	RRT-Connect	MEG-RRT*
Success Rate (SR)	100%	100%	100%	100%
Iterations to First, Solution ($I_{\mathrm{first}}$)				
Mean ± Std	1069 ± 490.4	994 ± 486.4	389 ± 154.1	406 ± 245.8
[Min, Max]	[272, 2446]	[379, 2327]	[136, 1164 ]	[116, 1408]
Computation Time ($T_{exec}$) [s]				
Mean ± Std	0.0065 ± 0.0049	0.0073 ± 0.0086	0.0015 ± 0.0016	0.3377 ± 0.1986
[Min, Max]	[0.0014, 0.0234]	[0.0017, 0.0815]	[0.0006, 0.0167]	[0.0871, 1.1711]
Final Path Cost ($C_{\mathrm{best}}$)				
Mean ± Std	86.6 ± 2.9	82.1 ± 2.9	113.6 ± 13.03	81.5 ± 2.3
[Min, Max]	[80.1, 90.3]	[79.3, 93.1]	[90.7, 174.1]	[78.8, 88.8]
Tree Size/Memory Efficiency ($N_{nodes}$)				
Mean ± Std	566 ± 311.7	520 ± 302.6	142 ± 47.1	512 ± 300.4
[Min, Max]	[156, 1503]	[171, 1335]	[77, 381]	[168, 1780]

## Data Availability

The original contributions presented in this study are included in the article. Further inquiries can be directed to the corresponding author.
